# A Virtual Ward Model of Care for Patients With COVID-19: Retrospective Single-Center Clinical Study

**DOI:** 10.2196/25518

**Published:** 2021-02-10

**Authors:** Olivia R Ferry, Emma C Moloney, Owen T Spratt, Gerald F M Whiting, Cameron J Bennett

**Affiliations:** 1 Metro North Hospital and Health Service Brisbane Australia

**Keywords:** COVID-19, efficacy, hospital, innovation, model, remote care, safety, telemedicine, virtual health care, virtual ward

## Abstract

**Background:**

COVID-19 has necessitated the implementation of innovative health care models in preparation for an influx of patients. A virtual ward model delivers clinical care remotely to patients in isolation. We report on an Australian cohort of patients with COVID-19 treated in a virtual ward.

**Objective:**

The aim of this study was to describe and evaluate the safety and efficacy of a virtual ward model of care for an Australian cohort of patients with COVID-19.

**Methods:**

Retrospective clinical assessment was performed for 223 patients with confirmed COVID-19 treated in a virtual ward in Brisbane, Australia, from March 25 to May 15, 2020. Statistical analysis was performed for variables associated with the length of stay and hospitalization.

**Results:**

Of 223 patients, 205 (92%) recovered without the need for escalation to hospital care. The median length of stay in the virtual ward was 8 days (range 1-44 days). In total, 18 (8%) patients were referred to hospital, of which 6 (33.3%) were discharged after assessment at the emergency department. Furthermore, 12 (5.4%) patients were admitted to hospital, of which 4 (33.3%) required supplemental oxygen and 2 (16.7%) required mechanical ventilation. No deaths were recorded. Factors associated with escalation to hospital care were the following: hypertension (odds ratio [OR] 3.6, 95% CI 1.28-9.87; *P*=.01), sputum production (OR 5.2, 95% CI 1.74-15.49; *P*=.001), and arthralgia (OR 3.8, 95% CI 1.21-11.71; *P*=.02) at illness onset and a polymerase chain reaction cycle threshold of ≤20 on a diagnostic nasopharyngeal swab (OR 5.0, 95% CI 1.25-19.63; *P*=.02).

**Conclusions:**

Our results suggest that a virtual ward model of care to treat patients with COVID-19 is safe and efficacious, and only a small number of patients would potentially require escalation to hospital care. Further studies are required to validate this model of care.

## Introduction

On March 11, 2020, the World Health Organization declared COVID-19, a respiratory infection due to SARS-CoV-2, as a global pandemic [1]. A key consideration in this pandemic has been the management of the rapid influx of patients with COVID-19. The subsequent strain on health care systems has acted as a catalyst for increasing the implementation of telemedicine [2]. Telemedicine refers to health care provision through information technologies and telecommunication systems [3]. A Cochrane review [4] concluded that telemedicine can have equivalent outcomes to those of in-person care. However, the implementation of novel telemedicine approaches can be challenging, since adaptation of both staff and patients is required. During COVID-19, telemedicine has been used to triage, treat, and coordinate care provision to patients with COVID-19 to improve health care access, reduce disease transmission, and optimize resource allocation [5-7]. A virtual ward delivers hospital-level care to patients in the community through telemedicine. Through the provision of timely multidisciplinary care, virtual ward models have reduced emergency department presentations and hospital admissions [8,9]. These outcomes are desirable in a pandemic, where the judicious use of limited health care resources is critical.

To provide care to patients safely and effectively through a virtual care model, it is important to understand the clinical course of COVID-19 [2]. Several meta-analyses of published cohort studies have described the most common initial symptoms of COVID-19, including cough and fatigue [10-12]. Common comorbidities identified in patients with confirmed COVID-19 are hypertension (15.6%), diabetes (7.7%), and cardiovascular disease (4.7%) [11]. The most common laboratory abnormalities include an increased C-reactive protein level (68.6%), lymphopenia (57.4%), and an increased lactate dehydrogenase level (51.6%) [12]. The reported clinical spectrum of COVID-19 is broad, ranging from asymptomatic infection and mild upper respiratory tract illnesses to severe pneumonia and critical multiorgan failure [13]. The current literature suggests that approximately 80% of cases are mild [13]. However, out of 44,500 cases of COVID-19 in China, 14% of patients experienced severe disease with hypoxia and 5% of critical cases experienced respiratory failure, shock, or multiorgan dysfunction [13]. Mortality rates vary by region and the data collection method. Initial studies in China have reported mortality rates of 2.3%-3.6%, with a higher mortality associated with a higher age or the presence of comorbidities [11-13]. This emphasizes the potential for most patients with COVID-19 to be treated in lower acuity settings with monitoring for disease exacerbation.

Predictors of disease exacerbation during acute COVID-19 have been proposed in early retrospective cohort studies on patients with COVID-19 pneumonia or severe disease [14-18]. Baseline characteristics such as increasing age, male sex, and comorbidities confer a greater risk of severe disease and mortality [14,17,19,20]. In particular, chronic lung disease, cardiovascular disease, hypertension, diabetes mellitus, and immunosuppression have been proposed as risk factors [21-23]. In severe disease, a higher incidence of dyspnea (approximately 67%) has been reported in those requiring admission to the intensive care unit (ICU) [14,18]. Additionally, new-onset dyspnea may reflect the development of COVID-19 pneumonia. In cohort studies on COVID-19 pneumonia, the median time to dyspnea onset has been reported as 5-8 days after initial symptom onset [15,16]. Additionally, high body temperatures (≤39°C) are associated with an increased likelihood of acute respiratory distress syndrome (ARDS) [17]. The time to deterioration is notable with a median of 8-12 days from illness onset to ARDS and 10 days to ICU admission [14-16,19]. While further data are needed, this second week of acute illness likely represents a high-risk period for disease exacerbation, which may bolster clinical decision making regarding hospitalization.

The aims of this study were as follows: (1) to describe the clinical characteristics of an Australian cohort of patients with COVID-19, (2) to evaluate the clinical care provided to this cohort through a virtual ward model, and (3) to identify any possible predictors of deterioration.

## Methods

### Study Design

A retrospective single-center clinical assessment was performed for patients admitted to the Metro North Virtual Ward from March 25 to May 15, 2020. This study was deemed at low/negligible risk by the Royal Brisbane and Women’s Hospital Human Research Ethics Committee. No formal power calculations were performed, owing to the inclusion of all patients meeting the study criteria.

### Study Population

All patients admitted to the virtual ward during the specified period were assessed in accordance with the following inclusion criterion: a laboratory-confirmed diagnosis of COVID-19 through polymerase chain reaction (PCR) detection of SARS-CoV-2 RNA on a diagnostic nasopharyngeal swab (NPS). Patients were excluded if they had a preliminary positive or inconclusive PCR result but a negative result on subsequent confirmatory PCR testing. Patients were admitted to the virtual ward from the community after notification of a positive PCR result by the Metro North Public Health Unit, Herston, Australia, or following hospital discharge, in cases of confirmed disease.

### Virtual Care

Patients remained in out-of-hospital isolation during their virtual ward admission with nursing observations obtained through telephonic consultations. Virtual ward staff were located in a secure dedicated hospital workspace with medical records maintained in accordance with local hospital procedures and protocols. Patients were risk-stratified by age, comorbidities, and symptom burden to determine the frequency of telephonic consultations: low-risk patients, once daily; high-risk patients, twice daily. Observations were structured to monitor patient symptoms and identify potential deterioration. During each consultation, patients were asked to rate (on a scale of none, mild, moderate, or severe) the following symptoms: shortness of breath, cough, fatigue, sputum production, nausea/vomiting, headache, myalgia, and sore throat. These symptoms were numerically scored at each review. Patients’ general well-being, social situation, and adherence to isolation were also assessed.

Clinical reviews were conducted by medical officers when the following prespecified escalation criteria were met: (1) the patient reported severe symptoms related to shortness of breath, cough, or fatigue; (2) symptoms became more severe either on 1 observation of patients aged >65 years and having comorbidities or over 2 observations in those without comorbidities; or (3) any staff or patient concerns regarding disease exacerbation. If required, hospital referral was arranged for further assessment. All patients were reviewed by a medical officer prior to discharge. Multidisciplinary care was provided, with pharmacists ensuring patient access to medications and social workers offering psychosocial support.

In accordance with the Communicable Diseases Network Australia (CDNA) COVID-19 guidelines [24], patients were discharged and released from self-isolation on meeting the following recovery criteria: (1) 10 days since symptom onset and resolution of all symptoms of acute illness in the past 72 hours and (2) 10 days since hospital discharge and resolution of all symptoms of acute illness in the past 72 hours, if hospitalized for severe COVID-19.

### Data Collection

Data on patient demographics, epidemiological history, comorbidities, medication history, COVID-19 symptoms, clinical reviews, pathology results, hospital assessment, and treatment outcomes were collected from existing medical records.

### Data Analysis

We expressed descriptive statistics as number (%) values for categorical data and median or mean (range) values for continuous variables. We performed Pearson *χ*^2^ tests to explore the risk factors among patients requiring hospital referral and those with a virtual ward stay >7 days. We used Fisher exact tests when event counts were <5. Missing data were not imputed in the analyses. Furthermore, we calculated odds ratio (OR) and corresponding 95% CI values. All tests were two-sided, with a *P* value <.05 considered significant. Data were not adjusted for multiple testing; hence, findings should be considered to be descriptive and should not be used to infer definitive effects. SPSS (version 26.0, IBM Corp) was used for the analyses.

## Results

### Patient Characteristics

**Figure 1:**
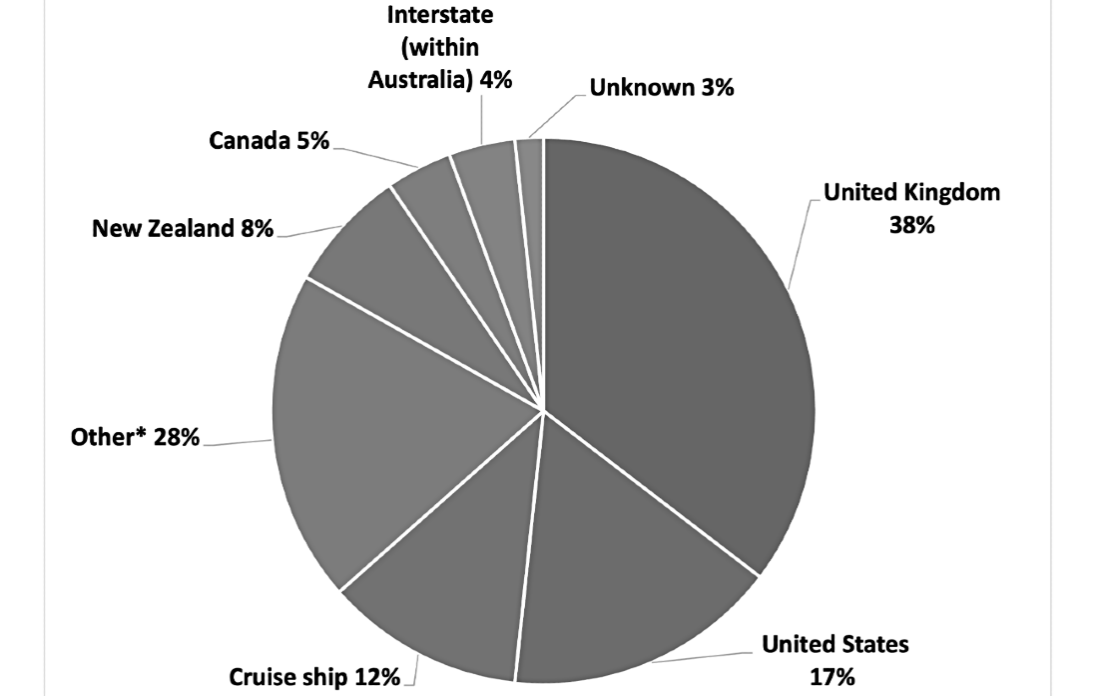
Cases epidemiologically linked to overseas or interstate travel (n=178). *Others: Argentina, Cuba, Egypt, Hong Kong, Indonesia, Japan, Myanmar, Norway, the Philippines, Singapore, South Africa, Sweden, Turkey, and the United Arab Emirates.

A total of 223 patients with a median age of 45 (range 14-78) years (female n=118, 52.9%) were assessed in this study (Table 1). This included 2 patients aged <18 years. Almost half (n=100, 44.8%) of the patients had a comorbidity, with hypertension (n=38, 17%) and asthma (n=24, 10.8%) being the most common manifestations. A total of 178 (79.8%) cases were epidemiologically linked to overseas travel (Figure 1), the most common destinations being the United Kingdom (n=68, 38%) and the United States (n=30, 17%). Furthermore, 16 (7.2%) patients had traveled on cruise ships. The most common COVID-19 symptoms upon presentation were cough (n=163, 73.1%), fever (n=117, 52.5%), and headache (n=103, 46.2%). Prior to virtual ward admission, 100 (44.8%) patients were assessed by a medical practitioner, either in person or through telemedicine, 21 (9.4%) had undergone chest radiography, and 22 (9.9%) had received laboratory blood tests. Initial diagnostic PCR detected SARS-CoV-2 RNA at a median cycle threshold (Ct) of 23.88.

**Table 1:** Baseline characteristics of the study population (N=223).

Characteristics		All patients	Patients not referred to hospital (n=205)	Patients referred to hospital (n=18)
Median age, years (range)		45.0 (14-78)	42.0 (14-78)	54.0 (23-71)
Female sex, n (%)		118 (52.9)	108 (52.7)	10 (55.6)
High risk^a^, n (%)		63 (28.3)	54 (26.3)	9 (50)
**Transmission source, n (%)**				
	Overseas travel	178 (79.8)	167 (81.5)	11 (61.1)
	Contact with a confirmed case	47 (21.1)	43 (21)	4 (22.2)
	Locally acquired	43 (19.3)	35 (17.1)	8 (44.4)
	Unknown	3 (1.3)	3 (1.5)	0 (0)
**Comorbidities, n (%)**				
	Any	100 (44.8)	87 (42.4)	13 (72.2)
	Hypertension	38 (17)	31 (15.1)	7 (38.9)
	Asthma	24 (10.8)	23 (11.2)	1 (5.6)
	Diabetes mellitus	13 (5.8)	12 (5.9)	1 (5.6)
	Immunosuppression^b^	6 (2.7)	5 (2.4)	1 (5.6)
	Chronic obstructive pulmonary disease	3 (1.3)	3 (1.5)	0 (0)
Medication (angiotensin-converting enzyme inhibitors angiotensin II receptor blockers, total 135), n (%)		25 (11.2)	19 (9.3)	6 (33.3)
**Initial symptoms at onset, n (%)**				
	Cough	163 (73.1)	149 (72.7)	14 (77.8)
	Fever	117 (52.5)	104 (50.7)	13 (72.2)
	Headache	103 (46.2)	97 (47.3)	6 (33.3)
	Sore throat	97 (43.5)	90 (43.9)	7 (38.9)
	Fatigue	84 (37.7)	74 (36.1)	10 (55.6)
	Rhinorrhea	82 (36.8)	76 (37.1)	6 (33.3)
	Myalgia	78 (35)	70 (34.1)	8 (44.4)
	Shortness of breath	52 (23.3)	46 (22.4)	6 (33.3)
	Nausea/vomiting	37 (16.6)	33 (16.1)	4 (22.2)
	Diarrhea	37 (16.6)	35 (17.1)	2 (11.1)
	Anosmia	36 (16.1)	35 (17.1)	1 (5.6)
	Ageusia	30 (13.5)	27 (13.2)	3 (16.7)
	Sputum	24 (10.8)	18 (8.8)	6 (33.3)
	Arthralgia	24 (10.8)	19 (9.3)	5 (27.8)
	Chest tightness	20 (9)	17 (8.3)	3 (16.7)
**Initial presentation**				
	Initial assessment by a medical practitioner, n (%)	100 (44.8)	90 (43.9)	10 (55.6)
	Initially admitted to hospital prior to virtual ward admission, n (%)	32 (14.3)	29 (14.1)	3 (16.7)
	Median time from symptom onset to initial nasopharyngeal swab (n=177), days (range)	4 (0-23)	4 (0-23)	1 (0-5)
	Median cycle threshold of polymerase chain reaction analysis of initial nasopharyngeal swabs (n=135) (range)	23.88 (11-36)	24.00 (11-36)	18.04 (14.6-33)
	Chest radiography, n (%)	21 (9.4)	20 (9.8)	1 (5.6)
	Blood tests, n (%)	22 (9.9)	21 (10.3)	2 (11.1)

^a^High risk was defined as age 65-85 years with any comorbidity or age 49-65 years with chronic lung disease, cardiovascular disease, immunosuppression, diabetes, or hypertension.

^b^Immunosuppression was defined as patients taking immunosuppressive medication or having a primary immunodeficiency.

### Virtual Ward Outcomes

Of the 223 patients in the virtual ward, 205 (92%) were discharged after clinical recovery without escalation to hospital care (Table 2). The median virtual ward admission length was 8 days (range 1-44 days). The median time to clinical recovery was 16 days (range 10-52 days). A total of 18 (8.1%) patients were referred for in-person hospital assessment (Table 3), of which 6 (33.3%) were assessed at the emergency department and discharged back to the virtual ward after review. The remaining 12 (66.7%) patients were admitted to an in-patient ward, of which 2 (16.7%) were admitted to the ICU and required mechanical ventilation (Table 3). The average length of hospital stay was 3.5 days (range 1-15 days) when admission to the ICU was not required. We recorded no mortality upon discharging the 223 patients assessed in this study.

Several factors were associated with a length of virtual ward stay >7 days. These included having any comorbidity (OR 2.0, 95% CI 1.15-3.40; *P*=.01), being classified as high risk on admission (OR 2.2, 95% CI 1.16-4.00; *P*=.02), or being hospitalized prior to virtual ward admission (OR 2.6, 95% CI 1.10-5.99; *P*=.03). Initial COVID-19 symptoms of cough (OR 2.2, 95% CI 1.22-4.10; *P*=.008), fevers or night sweats (OR 2.2, 95% CI 1.26-3.70; *P*=.005), and diarrhea (OR 2.3, 95% CI 1.06-5.07; *P*=.03) were also associated with a length of virtual ward stay >7 days.

**Table 2:** Virtual ward patient outcomes (N=223).

Outcome	Patients
Median length of stay, days (range)	8.0 (1-44)
Median time to clinical recovery^a^, days (range)	16 (10-52)
Discharged without complication, n (%)	205 (91.9)
Requiring hospital assessment, n (%)	18 (8.1)
Admitted to the in-patient ward, n (%)	12 (5.4)
Mean length of in-patient hospitalization, if admission to the intensive care unit was not required^a^, days (range)	3.5 (1-15)
Admitted to the intensive care unit, n (%)	2 (0.9)
Mortality, n (%)	0 (0)

^a^Time from symptom onset to clinical recovery in accordance with the Communicable Diseases Network Australia guidelines (at least 10 days since symptom onset and 72 hours of being asymptomatic).

**Table 3:** Clinical characteristics of patients requiring hospital care upon admission to the virtual ward (N=18).

Characteristics				Hospitalized patients
Median age, years (range)				54 (23-71)
Female sex, n (%)				10 (55.6)
High risk^a^, n (%)				9 (50)
Median day of illness upon referral to hospital, days (range)				8.50 (3-20)
Previous medical review on initial presentation, n (%)				10 (55.6)
Initial median polymerase chain reaction cycle threshold on diagnostic nasopharyngeal swab tests, n (range)				18.04 (14.61-33)
**Primary reason for referral, n (%)**				
	Shortness of breath		10 (55.6)	
	New or ongoing fevers		4 (22.2)	
	Chest pain or chest tightness		3 (16.7)	
**Hospital assessment on presentation**				
	Median oxygen saturation (n=13), % of ambient air (range)		96 (88-100)	
	Median heart rate (n=10), beats per minute (range)		80 (57-105)	
	Median respiratory rate (n=9), breaths per minute (range)		19 (16-28)	
	Fever (>37.5°C; n=11), n (%)		4 (22.2)	
	**Chest auscultation findings (n=15), n (%)**			
		Clear	9 (60)	
		Unilateral crackles	2 (13.3)	
		Bilateral crackles	4 (26.7)	
**Chest radiograph performed on presentation (n=15), n (%)**				
	No acute abnormality		8 (53.3)	
	Unilateral consolidation		2 (13.3)	
	Bilateral consolidation		2 (13.3)	
**Blood tests performed on presentation (n=16), n (%)**				
	Elevated lactate dehydrogenase (n=15)		9 (60)	
	Elevated C-reactive protein (n=6)		5 (83.3)	
	Lymphopenia (n=16)		4 (25)	
**Outcome, n (%)**				
	Assessed at the emergency department and discharged		6 (33.3)	
	Admitted to the in-patient ward		12 (66.7)	
	Prescribed antibiotics		8 (44.4)	
	Required supplemental oxygen upon admission		4 (22)	
	Admitted to the intensive care unit		2 (11.1)	
	Received mechanical ventilation		2 (11.1)	
	Mortality		0 (0)	
**Complications, n (%)**				
	Liver function derangement (n=16)		6 (37.5)	
	Respiratory failure		3 (16.7)	
	Acute kidney injury (n=13)		3 (23.1)	

^a^High risk was defined as age 65-85 years with any comorbidity or age 49-65 years with chronic lung disease, cardiovascular disease, immunosuppression, diabetes, or hypertension.

### Hospitalized Patients

In total, 18 patients with a median age of 54 (range 23-71) years (females: n=10 [55.6%]; high-risk: n=9 [50%]) were assessed in hospital upon virtual ward admission (Table 3). Referral to hospital occurred at a median of 8.5 days (range 3-20 days) since COVID-19 onset. Furthermore, 8 (43.9%) patients were reviewed by a medical officer prior to their virtual ward admission. Among 10 (55%) patients, the most common reason for care escalation was worsening, ongoing, or new-onset dyspnea. In addition, 4 (22%) patients were referred to hospital owing to new or ongoing fever, 3 (17%) for further clinical assessment of chest pain or chest tightness (Table 3), and the remaining 4 (23%) owing to a high symptom burden, functional decline with worsening fatigue, presyncopal symptoms, or suspected bacterial superinfection.

On hospital presentation, 4 (22%) patients had fever, and 4 (22%) had hypoxia and required supplemental oxygen on or shortly after presentation. Of the 18 patients presenting to hospital, 16 (88.9%) most commonly had an elevated lactate dehydrogenase level, liver function derangement, an elevated C-reactive protein level, or lymphopenia on blood tests. Furthermore, 15 (83.3%) patients underwent chest radiography, of which 4 (26.7%) had features of consolidation*.* Clinically, bacterial pneumonia was suspected in 7 (38.9%) hospitalized patients, and 8 (44.4%) patients were treated with antibiotics. Complications identified during hospitalization included respiratory failure in 3 (16.7%) patients, acute kidney injury in 3 (23.1%) patients, and liver function derangement in 6 (37.5%) patients. Moreover, 2 (11.1%) patients required admission to the ICU for mechanical ventilation.

Several possible predictors of deterioration associated with escalation of care were identified, including the presence of hypertension (OR 3.6, 95% CI 1.28-9.87; *P*=.01), sputum production at symptom onset (OR 5.2, 95% CI 1.74-15.49; *P=*.001), arthralgia at onset (OR 3.8, 95% CI 1.21-11.71; *P*=.02), and an PCR Ct for SARS-CoV-2 RNA of ≤20 on diagnostic NPS (OR 5.0, 95% CI 1.25-19.63; *P*=.02).

## Discussion

### Principal Findings

This retrospective study describes the characteristics and clinical course of an Australian cohort of patients with COVID-19 treated in a newly established virtual ward. To our knowledge, this is the first study to evaluate a community virtual ward model for patients with COVID-19 and the largest clinical assessment of patients with COVID-19 in Australia to date.

Epidemiologically, most cases were attributed to overseas travel, particularly to the United Kingdom, the United States, or cruise ships, consistent with a previous report from Australia [24]. In this cohort, cough, fever, and headache were the most common symptoms; however, their frequencies differed from those reported in a meta-analysis of hospitalized patients in China [10], thus potentially reflecting a lower disease severity in our cohort or reporting certain differences; our cohort recorded a higher incidence of sore throat (43.5% vs 11.6%) and rhinorrhea (36.8% vs 7.3%) and a lower incidence of chest tightness (9% vs 22.9%) and sputum expectoration (10.8% vs 23.7%) than those reported in the meta-analysis in China [10]. Approximately half of our patients had comorbidities, most commonly including hypertension (n=38, 17%) and asthma (n=24, 10.8%), comparable with a previous report [11].

Our results suggest that a virtual ward model is safe for patients with COVID-19. Overall, 205 (92%) patients recovered without escalation to hospital care. Furthermore, 18 (8.1%) patients required hospital assessment, of which only 12 (5.4%) were admitted to hospital and 2 (0.9%) were admitted to the ICU. This reflects a lower severity of COVID-19 in our cohort (0.9%) compared to that reported previously (5%) [15]. The mortality rate of 0 (0%) in our cohort is consistent with the low nationwide mortality rate of 1.3% in Australia [24], at the time of writing. The median virtual ward admission length was 8 days (range 1-44 days) with a median time to clinical recovery of 16 days (range 10-52 days). This discrepancy may be potentially attributed to returning overseas travelers who acquired the infection abroad. In the context of comorbidity and prior COVID-19–related hospitalization, higher-risk patients stayed longer in the virtual ward. These findings suggest that a virtual model of care can potentially preserve in-patient capacity and resources in hospitals and reduce the risk of disease transmission and hospital-acquired sequelae. Although most of our patients had mild illness, regular monitoring and supportive care may have reduced hospital presentations.

Timely identification of disease exacerbation is imperative for safe virtual care. Several studies have reported high diagnostic agreement between virtual and in-person consultations [25,26]. However, clinicians have cited concerns regarding telephonic consultations, primarily owing to the lack of physical examination [27]. COVID-19 has provided an opportunity to introduce a range of telemedicine approaches to the medical staff, owing to the necessity to deliver safe patient care during a pandemic. Remote assessment of dyspnea, the most common reason for hospital referral in our study, has been challenging during COVID-19 [28]. A difficulty in ruling out urgency upon telephonic consultations may have resulted in a lower threshold for hospital referral in our study, with one-third of hospital-referred patients subsequently discharged after in-person assessment [26]. Our virtual ward model was simple without monitoring equipment beyond household thermometers. Enhanced assessment capabilities through real-time telemonitoring may reduce diagnostic uncertainty [29].

Our hospital-referred patients had a higher median age of 54 (range 14-78) years, being referred to hospital at a median of 8.5 days (range 3-20 days) into their illness. Preexisting hypertension, a proposed risk factor for severe COVID-19, was associated with a 3.6-fold increase in hospitalization rates. Initial symptoms of sputum production and arthralgia were associated with hospital referral, which have not been previously reported. Patients with a PCR Ct for SARS-CoV-2 RNA of ≤20 on diagnostic NPS were 5-fold more likely to be referred to hospital. Although a lower Ct indicates a higher RNA sample quantity, the implication of this value for disease progression is unclear. Further studies are required to validate these findings.

The 2 (0.9%) patients admitted to the ICU had risk factors associated with severe COVID-19 [14]. These 2 men aged >60 years, with preexisting hypertension, presented disease exacerbation on day 10 of their illness and hence required ICU admission and ventilatory support; this is consistent with the reported median of 8-12 days to progression to ARDS and 10 days to ICU admission for patients with severe COVID-19 [14-16,19]. The pathogenesis of this late decline remains unclear; however, pathological hyperinflammation seems likely [30].

### Limitations

The limitations of this study include its observational design and retrospective data collection, which resulted in missing data across several variables reported herein. Few patients in our cohort had severe COVID-19. There may have been an ascertainment bias as patients with more severe COVID-19 may have been directly admitted to hospital for the duration of their illness, bypassing the virtual ward.

### Conclusions

To our knowledge, this is the largest cohort study of COVID-19 patients in Australia to be described to date and the first to evaluate a virtual ward model of care. This study provides evidence regarding the safety and feasibility of a virtual ward setting to treat patients with COVID-19. Further studies are needed to identify the early predictors of COVID-19 exacerbation and to validate this health care model.

## Abbreviations

ARDS: acute respiratory distress syndrome
CDNA: Communicable Diseases Network Australia
Ct: cycle threshold
ICU: intensive care unit
NPS: nasopharyngeal swab
OR: odds ratio
PCR: polymerase chain reaction
